# Temperature Effects on Biomass and Regeneration of Vegetation in a Geothermal Area

**DOI:** 10.3389/fpls.2017.00249

**Published:** 2017-03-07

**Authors:** Abdul Nishar, Martin K.-F. Bader, Eoin J. O’Gorman, Jieyu Deng, Barbara Breen, Sebastian Leuzinger

**Affiliations:** ^1^School of Sciences, Auckland University of TechnologyAuckland, New Zealand; ^2^Imperial College LondonLondon, UK

**Keywords:** global warming experiment, soil warming, *Kunzea tenuicaulis*, climate change, LASSO, Wairakei

## Abstract

Understanding the effects of increasing temperature is central in explaining the effects of climate change on vegetation. Here, we investigate how warming affects vegetation regeneration and root biomass and if there is an interactive effect of warming with other environmental variables. We also examine if geothermal warming effects on vegetation regeneration and root biomass can be used in climate change experiments. Monitoring plots were arranged in a grid across the study area to cover a range of soil temperatures. The plots were cleared of vegetation and root-free ingrowth cores were installed to assess above and below-ground regeneration rates. Temperature sensors were buried in the plots for continued soil temperature monitoring. Soil moisture, pH, and soil chemistry of the plots were also recorded. Data were analyzed using least absolute shrinkage and selection operator and linear regression to identify the environmental variable with the greatest influence on vegetation regeneration and root biomass. There was lower root biomass and slower vegetation regeneration in high temperature plots. Soil temperature was positively correlated with soil moisture and negatively correlated with soil pH. Iron and sulfate were present in the soil in the highest quantities compared to other measured soil chemicals and had a strong positive relationship with soil temperature. Our findings suggest that soil temperature had a major impact on root biomass and vegetation regeneration. In geothermal fields, vegetation establishment and growth can be restricted by low soil moisture, low soil pH, and an imbalance in soil chemistry. The correlation between soil moisture, pH, chemistry, and plant regeneration was chiefly driven by soil temperature. Soil temperature was negatively correlated to the distance from the geothermal features. Apart from characterizing plant regeneration on geothermal soils, this study further demonstrates a novel approach to global warming experiments, which could be particularly useful in low heat flow geothermal systems that more realistically mimic soil warming.

## Introduction

Soil temperature plays an important role in many of the abiotic and biotic processes that are integral to plant growth (Oelke and Zhang, 2004), above and below ground biomass (Abramoff and Finzi, 2014; Munir et al., 2015), plant productivity (Luo et al., 2009), nutrient uptake (Rustad et al., 2001), and diversity and distribution (Bond-Lamberty et al., 2006; Pickering and Green, 2009; Djebou and Singh, 2015). Changes in vegetation cover are a response resulting from both environmental and biological conditions. Several authors have reported significant relationships between temperature and vegetation indices (Zhang et al., 2004; Kumar and Shekhar, 2015; Wang et al., 2015). Moreover, soil temperature influences soil moisture levels and microbial function and productivity (Lukewille and Wright, 1997; Pregitzer and King, 2005).

It is generally found, based on field observations (Lapenis et al., 2014) and remotely-sensed data (Shen et al., 2014), that soil temperature levels vary widely across landscapes based on elevation (Balisky and Burton, 1995; Clinton, 2003) and climate (Kang et al., 2000). However, over the last 100 years, soil temperature has risen in many areas as a result of climate change (IPCC, 2013). The average global surface temperature increased by 0.74°C from 1906 to 2005 (IPCC, 2007) and most models predict a rise in global surface temperature of at least 1.5–2.0°C by the end of this century (IPCC, 2013). The increase in the surface temperature during the past century has contributed to changes in vegetation phenology, species ranges, and community composition (Walther, 2010; Villarreal and Jesus, 2012) and the projected global temperature increase will generally result in an increase in near-surface soil temperatures (Chapin and Körner, 1995; Oechel et al., 1995; Claussen et al., 1999; Betts, 2001; ACIA, 2005; Hinzman et al., 2005), affecting soil conditions (Rixen et al., 2008; Okkonen and Kløve, 2010) and vegetation structure, composition, and growth.

Warming experiments in the past have used a variety of heating methods, including electric heating (de Valpine and Harte, 2001), infrared radiation (Wan et al., 2002), reciprocal transplants (Jonasson et al., 1993), and open- and closed-top field greenhouses (Henry and Molau, 1997). These approaches obviously have their place and contribute to our understanding, but each of these methods come with their own set of limitations (Shaver et al., 2000). Geothermally-heated ecosystems have recently been identified as complementary natural warming experiments, where one can investigate long term adaptation of real-world communities across natural temperature gradients (O’Gorman et al., 2014). Typically, geothermal hotspots have been heated above ambient conditions for a very long time (Bibby et al., 1995). The levels of soil heat, steam, and gaseous output vary amongst geothermal systems (Legittimo and Martini, 1989; McGee, 1997), depending on geological structures, the depth of the magma chamber and the water table (Legittimo and Martini, 1989). The area of soil heat emissivity depends on the geothermal heat flow (Hoang, 2010) whereas the level of impact on vegetation is a function of the distance from the geothermal heat point source (Kershaw, 1985).

Although warming affects all plant life-cycle phases, plant regeneration has been suggested to be especially sensitive (Hedhly et al., 2009; Walck et al., 2011). Vegetation regeneration is a strong indicator of changes in soil conditions and an increase in soil temperature will have an adverse effect on vegetation regeneration levels (Althoff et al., 2016). Similarly, root biomass may change in response to altered environmental variables (Norby and Jackson, 2000). Soil temperature is a primary rate-regulating factor (Berg et al., 1993; Kirschbaum, 1995) and an increase in soil temperature may lead to an overall reduction in root biomass (Milchunas and Lauenroth, 2001; Carón et al., 2015).

In this study we analyzed the effect of geothermal warming on vegetation by assessing plant regeneration rates and root biomass across a wide range of soil temperatures and soil chemical properties. We specifically addressed the following questions: (i) How does soil warming affect vegetation regeneration and root biomass? (ii) What set of variables (temperature, soil chemistry, and their interactions) best predicts changes in below and above ground biomass? We hypothesized that vegetation regeneration and root biomass will show a negative correlation with increasing soil temperature. Additionally, we expected soil temperature to have a far greater impact on vegetation regeneration above- and below-ground than other environmental variables.

## Materials and Methods

### Study Area

The Taupo Volcanic Zone (TVZ) in the North Island of New Zealand covers an area of approximately 30 km × 150 km (Soengkono, 1995; Kissling and Weir, 2005), containing 23 stable and long-lived geothermal fields (Bibby et al., 1995) with varying heat outputs (Kissling and Weir, 2005). The Wairakei-Tauhara geothermal field in particular has distinctive assemblages of plants that survive under extreme geophysical and geochemical conditions (Given, 1980; Healy, 1992; Boothroyd and Stark, 2000; Death and Death, 2006). The section of the Wairakei-Tauhara geothermal field covered by this study is referred to as the Crown Road Geothermal Area (located at 38° 41′ 28.31″ S 176° 06′ 54.15″ E), covering an area of about 1 km^2^.

### Study Species

The plant species found in the study area and studied was *Kunzea tenuicaulis*, a shrub in the native tea tree genus *Kunzea* (Myrtaceae). *Kunzea tenuicaulis* propagates from seeds and is endemic to active geothermal sites and its growth habit is a good indicator of soil temperature and geothermally altered soil (Smale et al., 2009). Soil temperature has the largest influence on the distribution of *Kunzea tenuicaulis* (Given, 1980; Martin et al., 2000) with soil acidity and chemical concentrations having minor effects (Burns and Leathwick, 1995; Burns et al., 1995; Burns, 1997).

### Experimental Design

In order to determine the effects of soil temperature on above and below-ground vegetation regeneration, an experimental trial was implemented in December 2014. We delineated a grid within the study area, consisting of 18 150 m × 200 m blocks. We established a 0.6 m × 0.6 m plot within each block to span a range of surface temperature profiles, including three ambient plots (<19°C) and 15 plots in warm to hot areas (24–50°C). These subsurface spot temperature measurements were taken at a depth of 15 cm to assist with plot allocation.

We removed all vegetation, including roots, within our 0.6 m × 0.6 m plots, to allow regeneration and not just regrowth to take place. The 0.6 m × 0.6 m plots had 100% vegetation coverage before the experiment was set up. The vegetation-free plots were revisited on a monthly basis until December 2015 to monitor the aboveground regeneration rate as percentage cover within a 0.5 m × 0.5 m area, allowing for a 0.1 m buffer around the perimeter of each plot (Loetsch and Haller, 1973, München, Germany; Sachtler, 1975, Merida, Venezuela-Roma, Italy). The above ground vegetation regeneration was determined by the number of new seedlings found after 12 months.

We used the ingrowth core method to quantify root growth (Bledsoe et al., 1999; Milchunas and Lauenroth, 2001). Ingrowth cores consisted of wire cylinders (12 cm long, 3.5 cm diameter, 2 mm mesh size), containing root-free soil from a site within the geothermal area, with similar soil temperature. Three ingrowth cores were installed in each of the regeneration plots at the start of the experiment. We used a soil corer to create a cylindrical hole in each plot, inserting the ingrowth cores three cm below the soil surface, and covering them with topsoil. After excavation in December 2015, the cores were transferred to the lab where the new roots were separated from the soil with sieves with 1–3 mm mesh size, rinsed, and set in a water bath to be scanned. The images captured were analyzed using WinRHIZO software (Regent Instruments, Inc., Quebec, QC, Canada), which separated them into five size classes by diameter: 0–0.5 mm, 0.5–1 mm, 1–1.5 m, 1.5–3 mm, and 3–4.5 mm. The roots were measured using micrometer calipers and separated using tweezers. Once scanned, the roots were separated from water and dried at 70°C for 65 h (to constant dry weight) and weighed to determine the biomass. 65 h in the oven had removed all the moisture from root, any more time in the oven would have been redundant. Below ground biomass regeneration was determined by the combined biomass of all roots per plot.

### Environmental Variables

Instantaneous subsurface spot temperature measurements were used to determine the high temperature plot locations at the outset of the experiment. Instantaneous subsurface spot temperature was measured in all the blocks at a depth of 15 cm, using a Yokogawa TX10 digital thermometer (Yokogawa Electric Corporation, Musashino, Tokyo, Japan) connected to a type K thermocouple. Once the plots were selected, continuous soil temperature measurements were used to provide a detailed temperature profile throughout the 1-year duration of the experiment. For continuous soil temperature measurements, one Thermochron iButton (DS1921G) temperature logger (Maxim Integrated, San Jose, CA, USA) was buried in each of the 18 monitoring plots at a depth of 15 cm and left in place from December 2014 to December 2015. The data from the iButtons were retrieved on a monthly basis.

Soil moisture readings were taken in each of the 18 monitoring plots using a Decagon Devices ProCheck meter with a 10HS soil moisture sensor (Decagon Devices, Pullman, WA, USA). Soil was taken at the depth of 15 cm on a monthly basis (December 2014–December 2016). For soil pH analysis, a soil sample was taken from each of the plots and oven-dried. In July 2016, soil was removed using a soil corer from a depth of 15 cm. The oven-dried soil sample was mixed with deionised water (1:2.5 volumetric ratio of soil to deionised water) and was set for a day. After thorough mixing, each sample solution was measured using a pH meter.

Soil samples (100 g) were taken using a 15 cm × 3.5 cm (length × diameter) soil corer from each of the 18 plots, from 0 to 15 cm below the surface. The soil samples were taken during July 2015. In the lab, the samples were oven-dried at 60°C for 3 days and ground for testing. Soil samples were tested for sulfate (SO_4_^2–^), magnesium (Mg), potassium (K), iron (Fe^2+^ and Fe^3+^), calcium (Ca), phosphorus (P), manganese (Mn), boron (B), copper (Cu), nickel (Ni), lead (Pb), zinc (Zn), and cadmium (Cd) (Willett and Zarcinas, 1986; Franzen et al., 1999).

### Data Analysis and Model Selection

All statistical analyses and graphics were performed using R version 3.2.2. (R Core Team, 2013). Soil temperature fluctuations from December 2014–December 2015 were plotted to assist in assessing the effects of soil temperature on soil pH, soil moisture, soil chemicals, and vegetation regeneration. Principal component analysis (PCA) was used to test for relationships between the predictor variables: soil temperature, soil moisture, soil pH, and soil chemical levels. The relationships between variables were plotted using a biplot to aid visual interpretation. We performed a least absolute shrinkage and selection operator (LASSO) (package *lars*) regression to drop variables with coefficients of zero and reduce high dimensional data for the regression analysis and model selection. A simple linear regression analysis was run with each of the remaining predictor variables and either root biomass or vegetation regeneration as the response variable. Model selection was based on the Akaike’s Information Criterion (AIC). The model with the lowest AIC score was selected. Since vegetation regeneration was collected as proportion data, a logit transformation (package *logit*) was applied prior to the regression analysis.

## Results

### Soil Temperature

**FIGURE 1:**
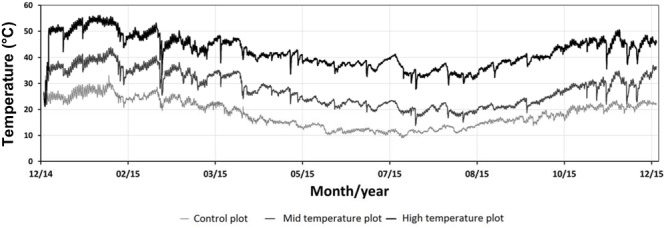
**Hourly soil temperature fluctuations at 15 cm depth (*n* = 18, control: *n* = 3, mid temperature: *n* = 11, high temperature: *n* = 4), December 2014–December 2015.** Data captured at Crown Road Geothermal Area.

**FIGURE 2:**
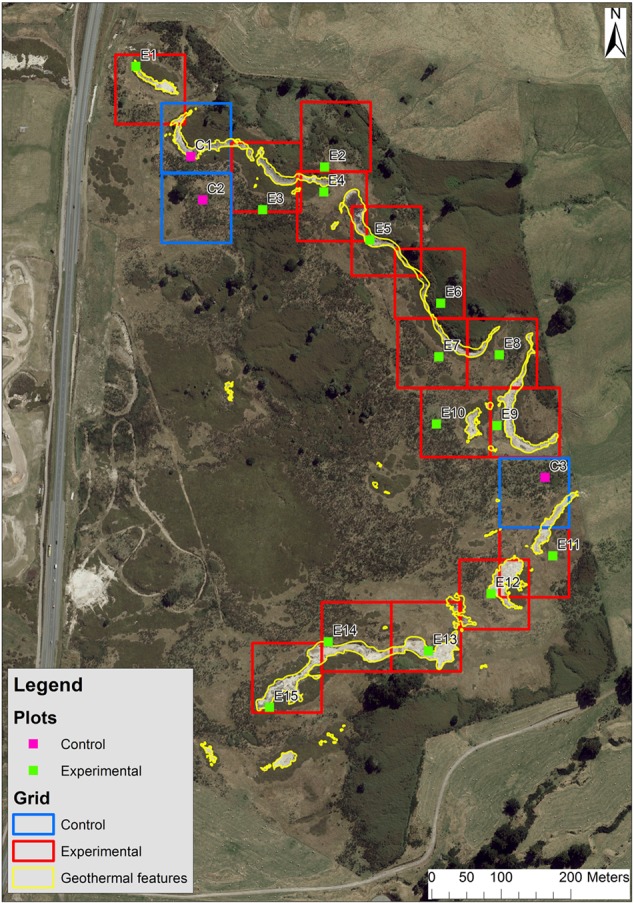
**Aerial photo of the study site, grid, and plot locations and geothermal features**.

The continuously logged soil temperature readings at the closest site to the geothermal features ranged between 18.5 and 70°C and the readings at the coolest, most distant site ranged between 6.5 and 32°C, from December 2014 to December 2015 (**Figure 1**). The soil temperature ranges of the high temperature plots were split into mid (18–43°C) and high (>56°C) temperate ranges. **Table 1** lists mean soil temperatures, while **Figure 2** shows the location of each plot with the study area.

**Table 1:** Experimental plots with mean soil temperatures over the study period.

Control plots	Mean soil temperature (°C)
C2	16.74
C3	17.19
C1	19.11
**Mid temperature plots**	
E3	19.33
E7	24.49
E4	25.65
E5	26.88
E6	27.36
E13	29.6
E9	30.56
E2	30.67
E15	36.05
E8	37.74
E11	37.93
**High temperature plots**	
E10	42.05
E12	45.72
E14	45.88
E1	50.43

### Soil Moisture and pH Value

**FIGURE 3:**
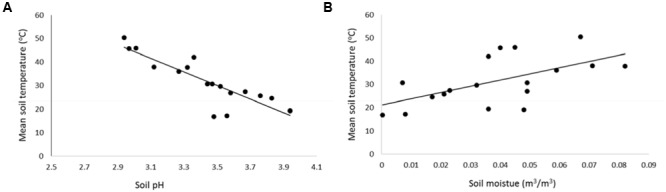
**Mean soil temperature at 15 cm depth (measured at the time of soil data capture) as a function of (A)** soil pH (*y* = -28.81*x*+ 130.84), and **(B)** volumetric soil moisture (*y* = 268.5*x*+ 21), *n* = 18. Data captured at 15 cm depth (*n* = 18, control: *n* = 3, mid temperature: *n* = 8, high temperature: *n* = 7), December 2014–December 2015. Data captured at Crown Road Geothermal Area.

Acidic soil occurred where soil temperatures were highest, and the soil pH increased in a linear fashion with a decline in soil temperature (**Figure 3**; *t*-value = 9.08, *P* < 0.001, *R*^2^ = 0.75). There was a significant increase in soil moisture with increasing soil temperature (**Figure 3**; *t*-value = 5.22, *P* = 0.006, *R*^2^ = 0.35).

### Soil Chemistry

**FIGURE 4:**
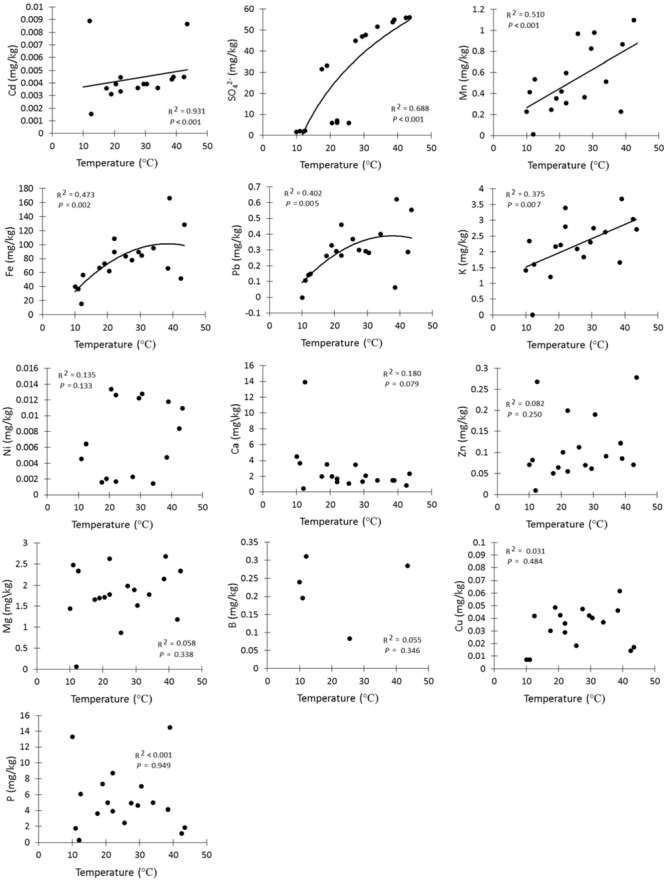
**Soil chemistry levels along a temperature gradient**.

**FIGURE 5:**
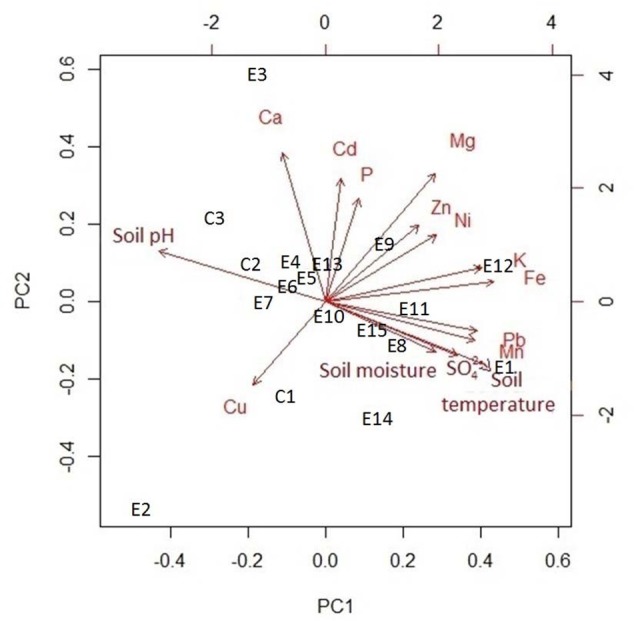
**Principal component analysis biplot of environmental variables for 18 plots**.

Amongst the major elements, iron (77.3 ± 0.05 mg kg^-1^, mean ± SE) and sulfate (28.3 ± 0.06 mg kg^-1^) had the highest concentrations in the soil samples (**Table 2**). The rarest trace element was manganese (0.6 ± 3.8 mg kg^-1^). In addition, lead (0.3 ± 12.5 mg kg^-1^, mean ± SE) and nickel (0.007 ± 538 mg kg^-1^) had the highest concentrations of the trace elements. The linear regressions (**Figure 4**) indicated a strong positive relationship between soil temperature and Cd, SO_4_^2–^, Mn, Fe, Pb, and K. However, there was no significant relationship detectable (**Figure 4**) between soil temperature and Ni, Ca, Zn, Mg, B, Cu, and P. The biplot (**Figure 5**) collectively displays the correlation of all the tested soil elements with soil temperature, moisture and pH; suggesting that soil temperature, soil moisture, SO_4_^2–^, Mn, Pb, K, and Fe are closely related while soil pH shows a negative correlation with temperature.

**Table 2:** Means and statistical parameters of major and minor soil elements from across the 18 plots, compared to control plots.

Element (mg kg^-1^)	Mean	Standard error	*t*-value	*P*
Ca	2.687	0.78	-1.88	0.086
Fe	77.29	0.05	3.79	0.005
K	2.216	2.37	3.1	0.013
Mg	1.787	3.88	0.99	0.367
Mn	0.615	3.79	4.08	0.003
P	5.306	0.68	-0.07	0.813
B	0.062	22.63	-0.97	0.342
SO_4_^2^**^-^**	28.28	0.06	5.94	<0.001
Cd	0.005	1186.53	0.09	0.976
Cu	0.041	70.94	-0.72	0.468
Ni	0.007	538.39	1.58	0.403
Pb	0.288	12.45	3.28	0.011
Zn	0.11	33.26	1.19	0.365

*Data captured at Crown Road Geothermal Area.*

### Vegetation Regeneration

**FIGURE 6:**
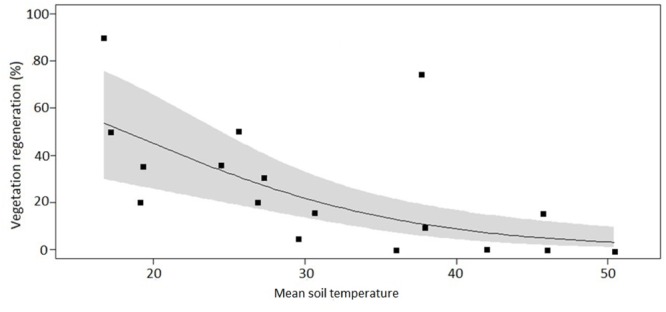
**Percentage vegetation regeneration cover across 18 plots spanning a soil temperature gradient at the Wairakei-Tauhara geothermal field**. The solid line indicates the fit of a linear regression model with a logit-transformed response variable (*y* = -1.45*x* + 71.5, *P* = 0.012, *R*^2^ = 0.34,). The gray-shaded area represents the 95% confidence interval.

After applying the LASSO regression with vegetation regeneration as the response variable, the coefficients of all but two explanatory variables were zero. Amongst these two candidates, the model containing soil temperature as sole predictor variable had the lowest AIC. The regeneration coverage at the time of assessment ranged from 0 to 90% in individual plots. On average, the highest regeneration of around 55% was observed at the coolest soil temperature (ca. 17°C) and declined in a curvilinear fashion with increasing soil temperature (*y* = -1.45*x* + 71.5, *P* = 0.012, *R*^2^ = 0.34, **Figure 6**). The control plots with mean temperatures ranging from 16.7 to 19.2°C had vegetation coverage between 50 and 90%. The intermediate plots with mean temperatures ranging from 20 to 38°C had vegetation coverage between 12 and 35% and the high temperature plots with mean temperature ranging from 42 to 50.4°C had vegetation coverage between 0 and 10%.

### Root Biomass

**FIGURE 7:**
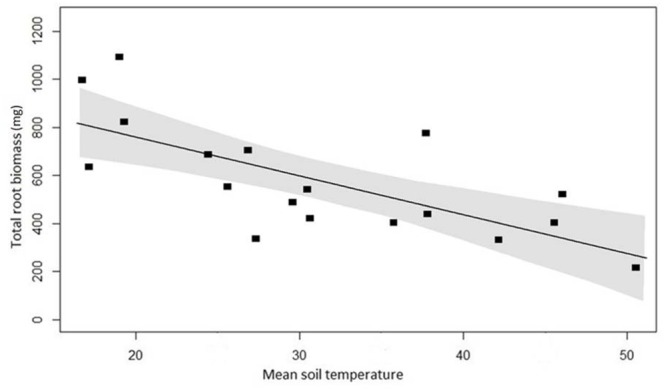
**Root biomass at 18 plots across the temperature gradient, with linear regression (*y* = -15.9*x*+ 1067.3, *P* < 0.001, *R*^2^ = 0.52).** The area between the gray-shaded area represents the 95% confidence interval.

The same LASSO model, applied to root biomass data, showed again that soil temperature as sole predictor variable yielded the lowest AIC. The variation in total biomass was strongly negatively related to soil temperature (**Figure 7**). Overall, there were 70% more roots regardless of root diameter in the cores excavated from the cooler control plots than plots with higher mean temperature. The difference in biomass between control and high temperature plots was greatest (243%) for fine roots (<1.5 mm) and decreased with increasing root diameter.

## Discussion

Understanding the impacts of soil warming on vegetation can help shape the conservation approaches of the future. This study, using geothermal heating, demonstrated the adverse effects of substantial soil warming on vegetation regeneration and root biomass. Our results indicate that after 1 year, two thirds of geothermally heated plots had vegetation regeneration of less than 35%, while all control (ambient) plots showed a minimum of 50% regeneration. Similarly, the control plots had significantly more biomass in roots of <0.5–3 mm in diameter.

Our analyses showed that soil temperature is the dominating effect on vegetation regeneration and root growth. Previous studies have also suggested that in geothermal fields, vegetation establishment and growth is strongly controlled by thermal gradients; (Burns, 1997; Elmarsdottir et al., 2003; Muukkonen, 2006; Chiarucci et al., 2008; van Manen and Reeves, 2012), having fundamental effects on the abiotic and biotic processes determining the distribution and density of geothermal vegetation (Chapin, 1983; Saito et al., 2009; Aalto et al., 2013; Olefeldt et al., 2013). Apart from growth rates and community composition, soil temperature may also affect species-specific growth forms as is the case with the dominant woody species in our study system (Harris, 1996; Boothroyd, 2009; Beadel et al., 2012).

In this study we have presented evidence that slow regeneration of above- and below-ground biomass is primarily due to elevated soil temperature. The observed regeneration rates in our high temperature plots suggest that vegetation regeneration will be slow in the case of soil warming but will not halt. Most importantly, we showed a consistent increase in root and vegetation growth in control plots and much slower regeneration in the high temperature plots. Our findings fully support our hypothesis that warming negatively affects root productivity and total biomass. The correlations of soil temperature with soil moisture, pH, and soil chemistry strongly suggest that soil warming will not only have a direct impact on vegetation by hindering development, but also indirectly by changing soil properties (Liu et al., 2015).

There was a positive relationship between soil temperature and soil moisture, which has also been shown in previous studies (Schmer and Werner, 1974; Idso et al., 1975; Pratt and Ellyett, 1979). This suggests that soil moisture content is geologically influenced in the same way as soil temperature (Legates et al., 2011; Wang et al., 2013). On the contrary, there was a negative relationship between soil temperature and soil pH. The lower pH together with high soil temperature is an indication of geothermal fluid and fume discharges (Sudarman et al., 2000) containing a range of trace elements (Rodríguez, 2014). Such low soil pH levels seen in the study area could negatively influence plant growth and biomass (Lakkaraju et al., 2010; Al-Traboulsi et al., 2013).

The mineral content of the geothermal fluid is absorbed by the organic matter and clay minerals in the soil, which are responsible for elevated concentrations of a variety of elements (Nicholson, 2012). The elements present in the soil as well as their concentration depends on the geothermal system (Van Kooten, 1987; Murray, 1997). Different geothermal systems have varying levels of crustal heat flow that are related to the presence of hot rocks located deeper in the crust (Rybach, 1981; DiPippo, 2005) and with the transfer of geothermal heat to the surface by the convection of ground water (Helgeson, 1968; Renner et al., 1975; Reiter et al., 1978), different concentrations of elements are transported to the surface (Mahon, 1970). Our soil chemistry analyses identified Fe and SO_4_^2–^ as the most abundant chemical species (mg/kg) amongst those tested. In a study of the Te Kopia Steamfield, New Zealand, Burns (1997) also noted high levels of extractable SO_4_^2–^ and Fe. Although Fe is one of the most abundant metals in the earth’s crust, its availability to plant roots is very low and it is largely driven by soil pH. At lower pH, Fe becomes more available for uptake by roots (Morrissey and Guerinot, 2009). Fe is essential for the plant’s metabolic processes but in excess amounts, it can be toxic (Aznar et al., 2015). Elevated amounts of SO_4_^2–^ in soil can have inhibitory effects on the growth, photosynthesis, and survival of plants (Ferguson and Lee, 1983; Austin and Wieder, 1987). Similar to SO_4_^2–^, the presence of trace elements may also be indicative of high temperature geothermal systems (Mahon, 1970; Brondi et al., 1973). Levels of Ca, Fe, K, Mg, Mn, P, B, Cd, Cu, Ni, Pb, and Zn found in this study environment are within the range reported for other geothermal systems (Ellis, 1970; Mahon, 1970).

## Conclusion

Our findings indicates that soil temperature is the main factor responsible for a decline in root biomass and vegetation regeneration rate. This study provides an important baseline for warming experiments at geothermal sites to track effects of changing temperate conditions on the vegetation community. This information is essential to better comprehend and forecast changes in the structure and composition of plant communities and develop adaptive management plans. In future studies aimed at using geothermal warming to understand the effect of changing climatic conditions, areas with very moderate warming (2–5°C) might be most suitable.

## Author Contributions

AN, SL, and BB conceived the idea. AN, SL, and MB developed the experimental design, AN and MB analyzed the data. JD conducted the soil chemistry data collection. AN wrote the manuscript, and all authors contributed to editing the final version.

## Conflict of Interest Statement

The authors declare that the research was conducted in the absence of any commercial or financial relationships that could be construed as a potential conflict of interest.
